# Factors Associated with Thoughts of Self-Harm or Suicide among Aboriginal and Torres Strait Islander People Presenting to Urban Primary Care: An Analysis of De-Identified Clinical Data

**DOI:** 10.3390/ijerph19010153

**Published:** 2021-12-23

**Authors:** Neha A. Pandeya, Philip J. Schluter, Geoffrey K. Spurling, Claudette Tyson, Noel E. Hayman, Deborah A. Askew

**Affiliations:** 1 General Practice Clinical Unit, School of Clinical Medicine, Royal Brisbane & Women’s Hospital, The University of Queensland, Herston, QLD 4029, Australia; n.pandeya@uq.edu.au (N.A.P.); philip.schluter@canterbury.ac.nz (P.J.S.); g.spurling@uq.edu.au (G.K.S.); noel.hayman@health.qld.gov.au (N.E.H.); 2 School of Health Sciences, University of Canterbury—Te Whare Wānanga o Waitaha, Christchurch 8140, New Zealand; 3 Southern Queensland Centre of Excellence in Aboriginal and Torres Strait Islander Primary Health Care, Metro South Health, P.O. Box 52, Inala, QLD 4077, Australia; claudette.tyson@health.qld.gov.au

**Keywords:** Aboriginal and Torres Strait Islander peoples, suicidal ideation, primary health care, epidemiological study

## Abstract

Suicide amongst Aboriginal and Torres Strait Islander people is a major cause of premature mortality and a significant contributor to the health and life expectancy gap. This study aimed to estimate the prevalence of thoughts of self-harm or suicide in Aboriginal and Torres Strait Islander people attending an urban primary health care service and identify factors associated with these thoughts. Multilevel mixed-effects modified Poisson regression models were employed to analyse three years of data gathered during the annual Aboriginal and Torres Strait Islander health assessments. At their first health assessment, 11.5% (191/1664) of people reported thoughts of suicide or self-harm in the prior two weeks. Having children, participating in sport or community activities or being employed full-time decreased the risk of such thoughts. Conversely, factors relating to social exclusion including homelessness, drug use, unemployment and job insecurity increased the risk of thoughts of self-harm or suicide. Individual clinicians, health services, and policy-makers all have a role in suicide prevention. Clinicians need appropriate training to be able to respond to people expressing these thoughts. Aboriginal and Torres Strait Islander community organisations need sovereignty and self-determination over resources to provide programs that promote cultural connectivity and address social exclusion, thereby saving lives.

## 1. Introduction

Aboriginal and Torres Strait Islander cultures are among the oldest surviving cultures in the world [1]. In pre-colonial times, and prior to the last 50 years, suicide amongst Aboriginal and Torres Strait Islander peoples was a rare occurrence [2]. Rates of suicide have increased since the 1980s, and it is now the fifth leading cause of death in this population [3].

Suicide amongst Indigenous peoples of colonised nations has been described as “one of a group of symptoms…that are in a large part interchangeable as expressions of the burden of grief and anger” in response to the profound and ongoing impacts of colonisation [4] (p. 96). These include the loss of land and culture, deliberate damage to family structures, discrimination, social disadvantage, denial of social justice and transgenerational trauma [2,5]. Furthermore, close kinship relationships mean that the impact of a loss through suicide is often compounded and extends beyond individual families and communities [6]. Suicide amongst Aboriginal and Torres Strait Islander peoples must, therefore, be acknowledged both in terms of personal circumstances that render individuals vulnerable, as well as in the context of the collective trauma that Aboriginal and Torres Strait Islander people continue to endure.

Suicide prevention strategies for Aboriginal and Torres Strait Islander populations have commonly focused on crisis-orientated services that recognise and respond to people ‘at risk’ [2]. Increasingly, a combination of three tiers of intervention is being recognised as essential, namely universal interventions aimed at the whole population; selective interventions for at-risk groups; and indicated interventions for at-risk individuals [7]. The first two tiers embody the Aboriginal definition of health where the health of an individual and their community are inextricably linked [8] and aim to heal and strengthen the social and emotional wellbeing of individuals and communities [7].

The social, cultural, political and historical determinants of health—inequalities and inequities in the circumstances and context in which people are born, grow, live and work—are key modifiable factors for influencing numerous health outcomes [9]. They include, but are not limited to, socioeconomic position, early childhood experiences, social support and exclusion, employment, housing, addiction, food security, access to transportation, and exposure to chronic stress [9]. These determinants often do not occur in isolation and have cumulative effects on health [10]. Addressing these determinants has been a key recommendation of numerous recent state and national suicide prevention strategies [11,12,13,14]. However, there remains relatively little research on the factors that are most relevant to suicidal behaviours in Aboriginal and Torres Strait Islander populations [6].

Suicidal behaviours (suicide, suicide attempt and deliberate self-harm) amongst Aboriginal and Torres Strait Islander peoples are associated with stressful life events, racism and discrimination, incarceration, unemployment, financial issues, alcohol and substance use, exposure to abuse, residing outside the family home, living in regional or remote areas, interpersonal and family conflict, mental illness, and psychological distress [15,16,17,18,19,20,21,22]. Conversely, good interpersonal relationships and participation in sport are associated with reduced suicidal behaviours [23]. ‘Cultural continuity’ factors, which protect community characteristics and help maintain a sense of cultural identity, have also been shown to be highly protective in other Indigenous populations internationally [24]. Marked differences in self-harm behaviours between communities with similar socioeconomic disadvantage, adverse childhood experiences and histories of colonisation, also suggest that there may be other community-specific factors at play [25]. Local-level data on risk and protective factors are thus essential to inform effective intervention strategies.

A contextual examination of suicidal ideation could provide a more complete picture of suicidal behaviours and are in themselves indicators of considerable emotional distress [26]. Similarly, whilst intentional self-injury is accepted as a separate construct, it is a strong predictive factor for suicide [27,28]. Heterogeneity in measurement tools, measurement intervals and study populations make general estimations on the prevalence of suicidal ideation in Aboriginal and Torres Strait Islander people impractical [15,17,18,19,20,21,23,29,30,31,32]. Moreover, these generalisations disregard the diversity of Aboriginal and Torres Strait Islander communities, and further highlight the need for community-specific data.

A cross-sectional analysis of annual Aboriginal and Torres Strait Islander health assessment data from 2014 to 2015 in an urban primary health care service showed that 8% of 780 Aboriginal and Torres Strait Islander adults reported thoughts of self-harm or suicide in the preceding two weeks [33]. This current study aimed to provide more recent prevalence data collected over a three-year period from Aboriginal and Torres Strait Islander people aged 15 years and over and to apply robust statistical modelling to identify social and cultural determinants of health that increased or decreased the risk of thoughts of self-harm or suicide in the same setting.

## 2. Methods

### 2.1. Study Design, Setting and Participants

This retrospective study used health assessment data from a cohort of Aboriginal and Torres Strait Islander adults aged ≥15 years who attended the Southern Queensland Centre of Excellence in Aboriginal and Torres Strait Islander Primary Health Care (Inala COE) between the 25 of July 2016 to 24 July 2019, inclusive. As of June 2019, the Inala COE had 2427 regular Indigenous adult clients [34]. The Inala COE is a Queensland government-run Aboriginal and Torres Strait Islander primary health care service, located in Inala, an outer south-western suburb of Brisbane, Australia [35]. Inala is on the traditional lands of the Yuggera people [36] and has one of the largest Aboriginal and Torres Strait Islander populations in Brisbane, with 5.5% of the population identifying as Aboriginal and/or Torres Strait Islander in the 2018 census [37].

Study participants had had at least one health assessment at the Inala COE during the study period. The first health assessment after 25 July 2016 defined entry into the cohort. Participants with an unknown or missing response for ethnicity were excluded.

### 2.2. Data Collection

The health assessment procedure at the Inala COE has been previously described [38]. In brief, clients are interviewed face-to-face using a structured format by a general practice nurse and a general practitioner. The health assessment includes demographics, physical health, lifestyle, living arrangements, employment, mental health and social engagement. Responses are inputted directly into a computerised form and are limited by pre-determined parameters including radio buttons, tick boxes, integers or numbers with defined decimal places. The completed form is uploaded into the patient’s medical record. Although data are collected primarily for clinical purposes, these forms have been purposefully designed to serve a dual clinical and research purpose, with responses also being automatically stored in a secure database for research purposes [38].

### 2.3. Study Variables and Definitions

The adapted Patient Health Questionnaire 9 (aPHQ-9) depression screening tool has formed a part of health assessments at the Inala COE since 2014. The aPHQ-9, based on the Patient Health Questionnaire 9, a reliable clinical and research tool used for diagnosing depressive disorders, has been adapted and validated for use with Aboriginal and Torres Strait Islander populations [39,40,41]. Item 9 of the aPHQ-9 questionnaire asks, ‘how often in the last 2 weeks have you been thinking about hurting or killing yourself?’. Responses were collapsed into two categories, no (‘none’) and yes (‘a little bit’, ‘most of the time’ and ‘all of the time’), to develop the primary outcome variable.

Predictor variables included age, sex, self-identified ethnicity, employment, education level, negative life events experienced within the previous 12 months, smoking, hazardous alcohol use, illicit substance use, history of incarceration, having children, being a single parent, being cared for by someone else (typically this applied to older people or those with a disability), participation in sport, engagement in physical exercise and participation in community or cultural activities within the previous 12 months. A full list of health assessment variables, prompts, available responses and analysed outcomes is presented in Table S1 in the Supplementary Materials.

### 2.4. Statistical Analysis

Reporting of analyses were informed by the REporting of studies Conducted using Observational Routinely collected health Data (RECORD) guidelines [42]. All analyses were performed using Stata SE version 17.0 (StataCorp, College Station, TX, USA), robust variance estimators were utilised for all regression models, and two-sided α = 0.05 defined statistical significance unless otherwise stated.

Initially, participant flow was described, together with health assessment visit distributions. Demographics were then presented for patients at their first health assessment within the study period and, using a χ^2^ test, those with one assessment were compared to those with two or more. Next, the distribution of the primary variable of interest, thoughts of self-harm or suicide, were presented and a binomial generalising estimating equation approach was used to assess whether there were changes in these rates over repeated health assessment visits. Bivariable analyses followed, investigating the relationship between thoughts of self-harm or suicide, demographic characteristics and a suite of potential associated factors. Conventionally employed logistic regression models produce odds ratios that are biased and inflated estimates of relative risks (RRs) when the outcome of interest is not rare [43], yet are commonly used and interpreted as such [44]. Therefore, a modified Poisson regression approach (with log-link function and robust variance estimators) was used to estimate RRs directly [44,45]. Multilevel mixed-effects modified Poisson regression models were thus employed for these bivariable and the subsequent multivariable analyses. In these models, repeated health assessment visit information was nested under participants, and participants were assumed to have random intercepts.

The development of the final multivariable model was guided by the recommendations of Sun and colleagues [46]. They advocate a backward elimination of variables after an initial bivariable screening. They suggest a *p*-value threshold for initial variable inclusion set at 0.15 to 0.25; a mid-range threshold of 0.20 was used here. They also suggest setting a *p*-value from 0.10 to 0.15 for deleting variables in the backward elimination modelling [46]. Due to the relatively large sample size, this was set here at 0.10. Because of the different patterns of missing data for each variable and, seeking to maximise available sample numbers at each iteration, a manual rather than automated backward elimination approach was undertaken. The Wald’s type III ꭓ^2^ statistic was used to determine *p*-values within these models, and the variable yielding the highest *p*-value above the 0.10 threshold was excluded. This was sequentially undertaken until all remaining variables within the model yielded a *p* ≤ 0.10. In order to maximise the number of authentic variables [46], a further stepwise procedure was undertaken whereby excluded variables were individually re-added to the derived parsimonious model and re-evaluated. This was repeated until no added variables resulted in a *p*-value ≤ 0.10, and all variables within the model met this criterion. This defined our “complete case multivariable model”. Once this model was derived, it was evaluated using the Hosmer–Lemeshow goodness-of-fit test, based on the conventionally employed ten partitions [47]. This was followed by an area under the receiver operating characteristic curve (AUC) analysis. AUC is frequently employed as a summary measure of a model’s predictive accuracy [48]. Adopting the recommendations of Hosmer and Lemeshow, an AUC of 0.5 suggests no discrimination, 0.7–0.8 is considered acceptable, 0.8–0.9 is considered excellent and more than 0.9 is considered outstanding [47]. Finally, a sensitivity analysis was conducted, using chained equations multiple imputation methods. This sensitivity analysis enabled all eligible health assessment records to be included within the derived multivariable model. Here M = 25 separate imputed datasets were generated and analysed. Aligned with the recommendations of Kontopantelis and colleagues [49], all variables considered in the bivariable models were used in this imputation strategy (including the primary variable of interest). However, analysis of the multiple imputation multivariable model only included those variables identified in the complete case selection strategies. This defined our “multiple imputation multivariable model”.

## 3. Results

### 3.1. Participants

A total of 3214 health assessments from 1893 participants aged ≥15 years were completed at the Inala COE during the study period. Of these, 131 assessments from 40 (2.1%) participants were excluded due to an ‘unknown’ or missing response for ethnicity, leaving 3083 health assessments from 1853 participants. A total of 991 (53.5%) participants had one health assessment, 526 (28.4%) had two, 304 (16.4%) had three and 32 (1.7%) had four assessments. The mean time between participants’ repeated health assessments was 13 months (range: 3 months, 58 months).

### 3.2. Demographics

Participants’ sociodemographic characteristics at their first health assessment within the study period are presented in Table 1. Most identified as an Aboriginal person, approximately 30% were in full-time or part-time employment, and just under 50% had a highest education level of Year 11 or higher—but a relatively high proportion of data (13.6%) were missing for this variable.

When comparing participants who had one health assessment to those with two or more, there were no differences by sex (*p* = 0.082), ethnicity (*p* = 0.202), education level (*p* = 0.058), or employment status (*p* = 0.273). However, a significant difference emerged by age (*p* < 0.001), with those aged from 15 to 24 years and 25–34 years having relatively fewer repeated health assessments (35.3% and 34.0%, respectively) than those aged 35–44 years, 45–54 years and ≥55 years (47.2%, 57.3%, and 64.9% respectively).

### 3.3. Thoughts of Self-Harm or Suicide

Valid data for the primary outcome measure, thoughts of self-harm or suicide within the prior 2 weeks, were available from 1664 (89.8%) participants at the first health assessment, 815 at the second, 313 at the third and 22 at the fourth. These thoughts were reported by 191 (11.5%) participants at the first assessment, 86 (10.6%) at the second, 29 (9.3%) at the third and 3 (12.0%) at the fourth health assessment. After accounting for the repeated measure nature of these data, these proportions were not significantly different over the various assessments (*p* = 0.621).

### 3.4. Demographic Factors Associated with Thoughts of Self-Harm or Suicide

Participants’ demographics were related to their thoughts of self-harm or suicide in the full sample using bivariable multilevel mixed-effects models, with multiple assessments nested under participants. These analyses revealed that sex (*p* = 0.008), age group (*p* < 0.001) and employment (*p* < 0.001) were significantly related to thoughts of self-harm or suicide, but ethnicity (*p* = 0.326) and education (*p* = 0.107) were not. To aid interpretation, these reports partitioned by participants’ demographics at their first health assessment within the study period are also presented in Table 1 and the estimated RRs and associated 95% CIs from these bivariable analyses appear in (1) of Table 2. Females had increased estimated RR compared to males, those aged younger than 55 years had higher rates than those aged ≥55 years, and those in full-time employment had lower RRs compared to their counterparts not employed full-time; see Table 1 and Table 2.

### 3.5. Potential Factors Associated with Thoughts of Self-Harm or Suicide

The frequency distributions of the potential factors related to thoughts of self-harm or suicide at participants’ first health assessments are presented in Supplementary Table S2. Bivariable multilevel mixed-effects analyses results appear in (1) of Table 2. Three variables had relatively high levels of missing data—having children, cared for by someone else, and being a single parent (46.5%, 39.7% and 37.7% missing respectively)—therefore a “missing” category was created and employed. Among the remaining potential factors, the median missing data percentage was 12.9% (Q_1_ = 3.4%, Q_3_ = 14.4%). In the bivariable analyses, all variables met the *p* ≤ 0.20 threshold for entering into the initial multivariable model except for ethnicity (*p* = 0.33), serious accident in the last 12 months (*p* = 0.70) and hazardous alcohol use (*p* = 0.98). Thus, these three variables were not considered within the multivariable model development.

### 3.6. Multivariable Multilevel Mixed-Effects Model Specification

The first phase of developing the multivariable multilevel mixed-effects model. Supplementary Table S3 includes results of the complete case model for all candidate variables prior to selection. Initially, backward elimination resulted in the elimination of 14 variables which had the highest *p*-values that exceeded 0.10—namely, in order: death of a family member or close friend in the previous 12 months, trouble with the police in previous 12 months, age group, witness to violence in previous 12 months, overcrowding at home, participation in exercise, family member in jail in previous 12 months, use of opiates, history of incarceration, being a single parent, experienced discrimination or racism in previous 12 months, education level, participation in sport and amphetamine use.

Next, each of the previously eliminated variables were sequentially re-added to the remaining variables in the model, and all variables were reinvestigated. The final complete case multivariable model included 13 variables and was based on 1627 (52.8%) observations from 1184 participants. The included variables and estimated RRs with associated 95% CIs from this model appears in (2) of Table 2.

It is evident from the complete case multivariable multilevel mixed-effects model that having children (RR = 0.50), and participation in sport (RR = 0.56) have relatively lower risks of thoughts of self-harm or suicide, while casual/contract employment (RR = 2.47), gambling problems (RR = 2.45) and homelessness (RR = 2.08) carry relatively higher risks; see Table 2. In terms of regression diagnostics, this final model yielded a Hosmer–Lemeshow goodness-of-fit *p* = 0.374 and the AUC = 0.749 (95% CI: 0.711, 0.787); a value which is considered acceptable. This evidence suggests that the final model has adequate fit.

### 3.7. Multiple Imputed Multivariable Model

Multiple imputations by chained equations (M = 25) were undertaken for missing data using all available variables. The created “missing” categories for the variables ‘being cared for by someone else’ and ‘being a single parent’ were reverted back to missing observations, whereas this category was retained for the variable ‘having children’—so that it could be directly compared to the complete case multivariable model. The estimated RR and associated 95% CIs from these analyses are presented in (3) of Table 2.

The concordance between the complete case (2) and imputed estimates (3) is generally high, with considerable overlap in the 95% CIs. However, in the multiple imputed multivariable model ‘participation in community activities’ was significant, and associated with a reduced likelihood of thoughts of suicide or self-harm (RR = 0.77, *p* = 0.046). The RR (and significance) for the variable ‘experiences of discrimination/racism <12 months’ increased from the imputed compared to the complete case analyses. For this multiple imputed model, the AUC = 0.752 (95% CI: 0.725, 0.778) which, again, is considered acceptable.

## 4. Discussion

Around one in nine (12%) of Aboriginal and Torres Strait Islander adults having health assessments at the Inala COE from 2016 to 2019 reported having thoughts of self-harm or suicide, an increase from the 8% reported at the same primary health care service four years earlier [33]. Having children, participating in sport or community activities, or being employed full-time were associated with a decreased probability of such thoughts. Conversely, being a casual or contract worker, being homeless or having a gambling problem more than doubled the risk of reported thoughts of self-harm or suicide. Being female, employed on a part-time basis, experiencing divorce or relationship breakdown, experiencing discrimination or racism, being unable to get a job, being a smoker or using cannabis also increased the risk of having these thoughts.

Cultural connectivity and cultural identity have been recognised as protective against suicide for Aboriginal and Torres Strait Islander peoples [6]. Our finding that participating in sport or community activities was associated with decreased thoughts of self-harm or suicide reflects the cultural strength of the Inala Aboriginal and Torres Strait Islander community [50], and the social connectivity and cultural affirmation attained through sporting activities in the community [51]. The protective nature of employment, and, conversely, the link between unemployment and the risk of suicidal ideation we identified have been well documented [2,6,7]. The perpetual cycle of unresolved grief and loss, which is consistently identified by Aboriginal and Torres Strait Islander communities as reducing social and emotional wellbeing [10], was not identified as a significant factor in our analysis. It is possible that the high prevalence of patients reporting the loss of a family member or a close friend within the last 12 months suggests the normalisation of grief and loss which may explain why this was not found to be a predictor in our study population. Similar to our findings, a recent systematic review of five studies that evaluated risk factors associated with suicide, self-harm and suicidal ideation identified the experience of racial discrimination as a common risk factor amongst Aboriginal and Torres Strait Islander youth [15].

This study has several strengths. To our knowledge, it is the first to use routinely collected clinical data, employing a multilevel mixed-effects approach, to analyse thoughts of self-harm or suicide amongst Aboriginal and Torres Strait Islander people aged 15 years and older, over a three-year period. Previous research was limited to a 12-month period [33] or focused on discrete age groups, particularly those aged 24 years or less [15]. The use of routinely collected health assessment data allowed for repeated measures without increasing participant burden. Compared to the existing literature, the study sample size was relatively large and covered a broader patient demographic with regards to age and sex. An additional strength of this study was the identification of social and cultural determinants of health associated with thoughts of self-harm or suicide—thus addressing an identified research gap [15]. Furthermore, the data were collected in a primary health care setting—the setting where the majority of mental health care is provided in Australia [52].

However, the generalisability of our findings may be limited owing to the use of health assessment data. Health assessments are often pre-booked appointments and often form a prerequisite to participating in local sporting and community activities, thus potentially including those who are more likely to engage in preventative health. Conversely, billing of the health assessment Medicare item enables access to Medicare-funded allied health services which may have biased patient inclusion towards those with chronic health conditions, a known risk factor for suicide [53]. Another limitation was missing data for item 9 regarding suicidal or self-harming thoughts in the aPHQ-9. Clinic staff may feel uncomfortable asking this question, or people may not wish to respond to this question. Finally, several community-level factors were unable to be measured owing to the constraints of using routinely collected clinical data.

Our findings have implications for clinicians and primary health care services. Firstly, dedicated programs to strengthen social and emotional wellbeing for groups more likely to experience suicidal thoughts need to be prioritised by health services, as does facilitation of access to specialised staff to ensure those experiencing homelessness or unemployment are supported to address these determinants. Secondly, as many community organisations are already aware, interventions for encouraging participation in sport and community activities generally should be considered in any suicide prevention strategies adopted by health services.

Individual clinicians, the health service, the community health sector and policy-makers all have a role to play in suicide prevention [7]. Aboriginal and Torres Strait Islander people are amongst the most socially excluded groups in Australia, being denied resources and rights in economic, social, political and cultural terms. Homelessness, drug use, unemployment and job insecurity are recognised consequences of social exclusion and in themselves continue to perpetuate the cycle of marginalisation and disadvantage experienced by many Aboriginal and Torres Strait Islander people. Unemployment and job insecurity are associated with poorer health outcomes and these negative health effects have been shown to be cumulative [9]. Commitment to Aboriginal and Torres Strait Islander workforce strategies remains a priority for suicide prevention, and this is even more pertinent in the context of the current COVID-19 pandemic [54]. In addition to being able to access culturally appropriate alcohol and drug services in primary care, Aboriginal and Torres Strait Islander people require access to a range of other culturally safe treatment options, including local residential withdrawal services, a need that has been repeatedly highlighted by the Inala community [55]. Secure and affordable housing should be a key priority for Aboriginal and Torres Strait Islander suicide prevention, with homelessness being a well-recognised risk factor for suicidal thoughts and behaviours [56].

Racism in all its forms must be confronted by policy-makers, health services and clinicians if suicidal and self-harming thoughts are to reduce. Accordingly, a fundamental aspect of reducing suicidal and self-harming thoughts and addressing the impacts of social exclusion will be further empowering Aboriginal and Torres Strait Islander community self-governance and control over the allocation of these resources.

## 5. Conclusions

Suicide prevention interventions amongst Aboriginal and Torres Strait Islander people need to be guided by an evidence base that identifies factors that positively or negatively impact the risk of suicide in Indigenous communities. Community-led and community-specific research is essential and the use of routinely collected health assessment data may make this more achievable for busy Aboriginal and Torres Strait Islander primary health care organisations. Chart audits exploring the outcomes for people who voiced suicidal ideation at their health assessment may also provide invaluable data for informing local suicide prevention pathways. Although outside the scope of health assessments, focused studies on the role of racism and community engagement using multi-item validated questionnaires or qualitative study design would help further elucidate relationships between these factors and suicidal and self-harming thoughts.

## Figures and Tables

**Table 1:** Distribution of patient sociodemographic characteristics at their first health assessment within the study period, overall and partitioned by thoughts of self-harm or suicide.

			Thoughts of Self-Harm or Suicide < 2 Weeks ^f^			
	Overall		No		Yes	
	*n*	(%)	*n*	(%)	*n*	(%)
Sex ^a^						
Female	937	(50.6)	722	(83.4)	114	(13.6)
Male	914	(49.4)	749	(90.7)	77	(9.3)
Age group (years)						
15–24	521	(28.1)	416	(84.6)	76	(15.4)
25–34	335	(18.1)	287	(91.1)	28	(8.9)
35–44	320	(17.3)	267	(89.9)	30	(10.1)
45–54	344	(18.6)	288	(87.0)	43	(13.0)
≥55	333	(18.0)	215	(93.9)	14	(6.1)
Ethnicity						
Aboriginal	1691	(91.3)	1343	(88.5)	175	(11.5)
Torres Strait Islander	60	(3.2)	45	(91.8)	4	(8.2)
Both ^b^	102	(5.5)	85	(87.6)	12	(12.4)
Education level ^c^						
Year 10 or less	807	(50.4)	636	(90.3)	68	(9.7)
Year 11 or more	794	(49.6)	657	(88.1)	89	(11.9)
Employment ^d^						
Full-time employed	415	(22.7)	354	(93.9)	23	(6.1)
Part-time employed	125	(6.8)	101	(87.8)	14	(12.2)
Casual/contract only	62	(3.4)	52	(91.2)	5	(8.8)
Other ^e^	1228	(67.1)	950	(86.4)	149	(13.6)

Note: ^a^ 2 (0.1%) values missing; ^b^ Both represents Aboriginal and Torres Strait Islander; ^c^ 252 (13.6%) values missing; ^d^ 23 (1.2%) values missing; ^e^ Other includes unemployed, pension, studying, pair carer; ^f^ 189 (10.2%) values missing.

**Table 2:** (1) Bivariable, (2) complete case multivariable (N = 1627; 52.8%), and (3) multiple imputation multivariable (N = 3083; M = 25) estimated relative risk (RR) and associated 95% confidence intervals (CIs) of thoughts of self-harm or suicide derived from multilevel mixed-effects models with repeated assessments nested under patients.

	(1) Bivariable		(2) Complete Case Multivariable		(3) Multiple Imputation Multivariable	
	RR (95% CI)	*p*	RR (95% CI)	*p*	RR (95% CI)	*p*
Sex		0.008		0.042		0.001
Female	1.39 (1.09, 1.77)		1.40 (1.01, 1.94)		1.48 (1.16, 1.88)	
Male	1 (reference)		1 (reference)		1 (reference)	
Age group (years)		<0.001				
15–24	2.67 (1.79, 3.98)					
25–34	2.01 (1.28, 3.15)					
35–44	1.63 (1.01, 2.63)					
45–54	2.34 (1.55, 3.54)					
≥55	1 (reference)					
Ethnic identification		0.326				
Aboriginal	1 (reference)					
Torres Strait Islander	0.66 (0.27, 1.62)					
Both ^a^	1.33 (0.82, 2.15)					
Education		0.107		0.039		0.017
Year 10 or less	1 (reference)		1 (reference)		1 (reference)	
Year 11 or more	1.24 (0.95, 1.61)		1.42 (1.02, 1.99)		1.36 (1.06, 1.76)	
Employment		<0.001		0.014		<0.001
Full-time employed	1 (reference)		1 (reference)		1 (reference)	
Part-time employed	2.23 (1.26, 3.92)		1.99 (1.05, 3.74)		1.81 (1.04, 3.16)	
Casual/contract only	2.89 (1.55, 5.39)		2.47 (1.27, 4.82)		2.34 (1.32, 4.15)	
Other ^b^	2.50 (1.72, 3.63)		1.20 (0.72, 2.00)		1.65 (1.10, 2.49)	
Homelessness		<0.001		0.003		0.001
No	1 (reference)		1 (reference)		1 (reference)	
Yes	3.61 (2.61, 4.98)		2.08 (1.28, 3.39)		1.95 (1.34, 2.86)	
History of incarceration		0.011				
No	1 (reference)					
Yes	1.42 (1.08, 1.86)					
Serious accident < 12 months		0.701				
No	1 (reference)					
Yes	0.88 (0.47, 1.67)					
Death of family/close friend < 12 months		0.054				
No	1 (reference)					
Yes	1.26 (1.00, 1.59)					
Discrimination/racism < 12 months		<0.001		0.078		<0.001
No	1 (reference)		1 (reference)		1 (reference)	
Yes	1.71 (1.29, 2.27)		1.37 (0.97, 1.95)		1.65 (1.27, 2.15)	
Divorce/separation < 12 months		<0.001		0.018		0.011
No	1 (reference)		1 (reference)		1 (reference)	
Yes	1.89 (1.42, 2.50)		1.54 (1.08, 2.20)		1.46 (1.09, 1.95)	
Gambling problems < 12 months		0.005		0.003		0.014
No	1 (reference)		1 (reference)		1 (reference)	
Yes	2.10 (1.25, 3.53)		2.45 (1.35, 4.45)		1.90 (1.14, 3.18)	
Family member sent to/in jail < 12 months		0.003				
No	1 (reference)					
Yes	1.44 (1.13, 1.85)					
Difficulty getting a job < 12 months		<0.001		0.032		0.002
No	1 (reference)		1 (reference)		1 (reference)	
Yes	2.27 (1.76, 2.92)		1.53 (1.04, 2.25)		1.58 (1.18, 2.12)	
In trouble with police < 12 months		0.002				
No	1 (reference)					
Yes	1.58 (1.18, 2.11)					
Overcrowding at home < 12 months		0.029				
No	1 (reference)					
Yes	1.51 (1.04, 2.19)					
Witness to violence < 12 months		<0.001				
No	1 (reference)					
Yes	1.98 (1.55, 2.53)					
Hazardous alcohol use		0.979				
No	1 (reference)					
Yes	1.00 (0.80, 1.25)					
Smoking		<0.001		0.021		0.004
Non-smoker ^c^	1 (reference)		1 (reference)		1 (reference)	
Current smoker	2.07 (1.60, 2.67)		1.55 (1.07, 2.24)		1.48 (1.13, 1.95)	
Amphetamine use		<0.001				
No	1 (reference)					
Yes	2.59 (1.92, 3.48)					
Cannabis use		<0.001		0.027		0.015
No	1 (reference)		1 (reference)		1 (reference)	
Yes	2.18 (1.74, 2.73)		1.47 (1.04, 2.06)		1.35 (1.06, 1.71)	
Opiate use		0.005				
No	1 (reference)					
Yes	1.86 (1.20, 2.87)					
Cared for by someone else ^d^		0.004				
No	1 (reference)					
Yes	1.32 (0.90, 1.92)					
Missing	0.73 (0.60, 0.93)					
Children ^e^		<0.001		<0.001		<0.001
No	1 (reference)		1 (reference)		1 (reference)	
Yes	0.63 (0.47, 0.83)		0.50 (0.35, 0.72)		0.53 (0.40, 0.70)	
Missing	0.49 (0.37, 0.64)		0.45 (0.30, 0.66)		0.51 (0.39, 0.67)	
Single parent ^f^		<0.001				
No	1 (reference)					
Yes	1.10 (0.82, 1.47)					
Missing	0.61 (0.47, 0.79)					
Community activity participation < 12 mths		<0.001		0.058		0.046
No	1 (reference)		1 (reference)		1 (reference)	
Yes	0.64 (0.50, 0.82)		0.71 (0.49, 1.01)		0.77 (0.59, 1.00)	
Exercise		0.006				
No	1 (reference)					
Yes	0.74 (0.59, 0.91)					
Participation in sport		0.015		0.035		0.061
No	1 (reference)		1 (reference)		1 (reference)	
Yes	0.63 (0.44, 0.91)		0.56 (0.33, 0.96)		0.71 (0.49, 1.02)	

Note: ^a^ Both represents Aboriginal and Torres Strait Islander; ^b^ Other includes unemployed, pension, studying, paid carer; ^c^ Includes ex-smokers; ^d^ 1224 (39.7%) values missing; ^e^ 1433 (46.5%) values missing; ^f^ 1163 (37.7%) values missing.

## Data Availability

The data that support the findings of this study are not publicly available, but are available from the Inala COE, via the corresponding author, providing appropriate ethical and community approvals are obtained.

## Supplementary Materials

The following are available online at https://www.mdpi.com/article/10.3390/ijerph19010153/s1, Table S1: Summary of health assessment prompts, responses and the outcome analysed; Table S2: Distribution of patient sociodemographics and potential factors at their first health assessment within the study period, overall and partitioned by thoughts of self-harm or suicide; Table S3. (1) Bivariable, complete case multivariable—without variable selection (*N* = 1240; 40.2%), (2) complete case multivariable (*N* = 1627; 52.8%) and (3) multiple imputation multivariable (*N* = 3083; M = 25) estimated relative risk (RR) and associated 95% confidence intervals (CIs) of thoughts of self-harm or suicide derived from multilevel mixed-effects models with repeated assessments nested under patients.
