# Supplementary material for: Factors Associated with Thoughts of Self-Harm or Suicide among Aboriginal and Torres Strait Islander People Presenting to Urban Primary Care: An Analysis of De-Identified Clinical Data
Source: Int J Environ Res Public Health. 2021 Dec 23;19(1):153. doi: 10.3390/ijerph19010153 (PMC8750353; doi:10.3390/ijerph19010153)
Supplement: Supplementary file 1 [file ijerph-19-00153-s001.zip › ijerph-1504229-supplementary.pdf]

Supplementary Materials

Table S1. Summary of Health Assessment prompts, responses and the outcome analysed.

Explanatory Variable	Health Assessment Prompt	Health Assessment Responses	Outcome Analysed
Sex	Sex auto-populates from client's medical record	Male, Female, Missing	Male, Female
Age	Age is automatically calculated by subtracting the date of birth from the date of the visit when the Health Assessment is completed	Age	15–24 years, 25–34 years, 35–44 years, 55 years and above
Ethnicity	Ethnicity	Aboriginal, Aboriginal & Torres Strait Islander, Torres Strait Islander, Other, Missing	Aboriginal, Aboriginal & Torres Strait Islander,<br>Torres Strait Islander
Education	Level completed	Year 10 or less, Year 11–12, TAFE, University, Not stated, missing	Year 10 or less (year 10 or less),<br>Year 11 or more (year 11–12, TAFE, university)
Smoking	Never smoked<br>Ex-smoker<br>Current smoker	Yes, No, Missing<br>Yes, No, Missing<br>Yes, No, Missing	Current smoker, Non-smoker, Missing
Alcohol	How often do you have a drink containing alcohol?<br>When you have a drink, how many do you usually have in one day?<br>How often do you have six or more drinks in one day?	AUDIT-C [1] score calculated from health assessment prompts, Missing	Yes (if AUDIT-C ≥ 3 for women AUDIT-C ≥ 4 for men) , No (if AUDIT-C < 3 for women AUDIT-C < 4 for men), Missing
Substance use	Other drugs	Yes, No, Missing	Not analysed independently
	Opiates (heroin, methadone, codeine, endone, MS contin)	Yes, No, Missing	Yes, No (if opiates = 'no' and other drugs = 'no'),<br>Missing
	Cannabis/Yarndi	Yes, No, Missing	Yes, No (if cannabis/yarndi = 'no' and other drugs = 'no'), Missing
	Amphetamines (speed, base, crystal meth, ice, ecstasy, MDMA)	Yes, No, Missing	Yes, No (if amphetamines = 'no' and other drugs = 'no'), Missing
Exercise	How many days per week do you do 30 minutes of huffing and puffing physical activity?	0–7	0 days per week, ≥1 days per week, Missing
Participation in a regular sport	Do you play any regular sport?	Yes, No, Missing	Yes, No, Missing
Incarceration	Incarceration history	Yes, No, Missing	Yes, No, Missing
Carer status	Number of children	Number of children, Missing	0 children, ≥1 child, Missing
	Are you a single parent?	Yes, No, Missing	Yes, No, Missing
	Are you cared for by someone else	Yes, No, Missing	Yes, No, Missing
Employment	Unemployed	Yes, No, Missing	

	Employed full-time<br>employed part-time<br>Casual<br>Contract work<br>Voluntary work<br>Full-time study<br>Part-time study<br>Home duties<br>Disability pension<br>Other pension	Yes, No, Missing<br>Yes, No, Missing<br>Yes, No, Missing<br>Yes, No, Missing<br>Yes, No, Missing<br>Yes, No, Missing<br>Yes, No, Missing<br>Yes, No, Missing<br>Yes, No, Missing<br>Yes, No, Missing	Full-time employed (if employed full-time = 'yes')<br>Part-time employed (if employed part-time = 'yes' and employed full-time ≠ 'yes')<br>Casual/contract only (if employed casual = 'yes' or employed contract = 'yes' and employed full-time or part-time ≠ 'yes' and studying full-time or part-time ≠ 'yes')<br>Other (if employed full-time or part-time ≠ 'yes' and employed casual or contract ≠ 'yes' and at least one of the remaining variables was indicated with 'yes')
Homelessness	Homelessness	Yes, No, Missing	Yes, No, Missing
Adapted Negative Life Event Scale (NLES)[2]	Have you experienced any of the following in the last 12 months?		
	Serious accident?	Yes, No, Missing	Yes, No (if serious accident = 'no' or serious accident = 'missing' and no reported stressors = 'yes'), Missing
	Death of a family member or close friend?	Yes, No, Missing	Yes, No (if death of a family member or close friend = 'no' or death of a family member or close friend = 'missing' and no reported stressors = 'yes'), Missing
	Divorce or separation?	Yes, No, Missing	Yes, No (if divorce or separation='no' or divorce or separation='no' and no reported stressors='yes'), Missing
	Not able to get a job?	Yes, No, Missing	Yes, No (if not able to get a job = 'no' or not able to get a job = 'no' and no reported stressors = 'yes'), Missing
	Witness to violence?	Yes, No, Missing	Yes, No (if witness to violence = 'no' or witness to violence = 'no' and no reported stressors = 'yes'), Missing
	Trouble with the police?	Yes, No, Missing	Yes, No (if trouble with the police = 'no' or trouble with the police = 'no' and no reported stressors = 'yes'), Missing
	Gambling problems?	Yes, No, Missing	Yes, No (if gambling problems = 'no' or gambling problems = 'no' and no reported stressors = 'yes'), Missing
	Member of family sent to jail / currently in jail?	Yes, No, Missing	Yes, No (if member of family sent to jail / currently in jail ='no' or member of family sent to jail / currently in jail = 'no' and no reported stressors = 'yes'), Missing
	Overcrowding at home?	Yes, No, Missing	Yes, No (if overcrowding = 'no' or overcrowding = 'no' and no reported stressors = 'yes'), Missing
	Racism or discrimination	Yes, No, Missing	Yes, No (if racism or discrimination = 'no' or racism or discrimination = 'no' and no reported stressors = 'yes'), Missing
	No reported stressors?	Yes, No, Missing	Not analysed independently
Community involvement	Have you participated in a community or cultural activity within the last 12 months	Yes, No, Missing	Yes, No, Missing

Table S2. Distribution of patient demographics and potential factors at their first Health Assessment within the study period, overall and partitioned by thoughts of self-harm or suicide.

	Overall		Thoughts of Self-Harm or Suicide < 2weeks			
			No		Yes	
	n	(%)	*n*	(%)	*n*	(%)
Sex						
Female	937	(50.6)	722	(83.4)	114	(13.6)
Male	914	(49.4)	749	(90.7)	77	(9.3)
Age group (years)						
15–24	521	(28.1)	416	(84.6)	76	(15.4)
25–34	335	(18.1)	287	(91.1)	28	(8.9)
35–44	320	(17.3)	267	(89.9)	30	(10.1)
45–54	344	(18.6)	288	(87.0)	43	(13.0)
≥55	333	(18.0)	215	(93.9)	14	(6.1)
Ethnic identification						
Aboriginal	1691	(91.3)	1343	(88.5)	175	(11.5)
Torres Strait Islander	60	(3.2)	45	(91.8)	4	(8.2)
Both [a]	102	(5.5)	85	(87.6)	12	(12.4)
Education level						
Year 10 of less	807	(50.4)	636	(90.3)	68	(9.7)
Year 11 or more	794	(49.6)	657	(88.1)	89	(11.9)
Employment						
Full-time employed	415	(22.7)	354	(93.9)	23	(6.1)
Part-time employed	125	(6.8)	101	(87.8)	14	(12.2)
Causal/contract only	62	(3.4)	52	(91.2)	5	(8.8)
Other [b]	1228	(67.1)	950	(86.4)	149	(13.6)
Homelessness						
No	1487	(95.8)	1213	(90.1)	133	(9.9)
Yes	65	(4.2)	36	(58.1)	26	(41.9)
History of incarceration						
No	1474	(84.5)	1194	(89.7)	137	(10.3)
Yes	270	(15.5)	210	(83.7)	41	(16.3)
Serious accident <12 months						
No	1534	(95.5)	1245	(89.2)	150	(10.8)
Yes	72	(4.5)	63	(91.3)	6	(8.7)
Death of family/close friend <12 months						
No	966	(59.5)	795	(90.3)	85	(9.7)
Yes	657	(40.5)	524	(87.0)	78	(13.0)
Discrimination/racism <12 months						
No	1350	(85.5)	1108	(90.7)	114	(9.3)
Yes	229	(14.5)	179	(82.5)	38	(17.5)
Divorce/separation <12 months						
No	1412	(87.7)	1149	(89.8)	131	(10.2)
Yes	198	(12.3)	159	(85.5)	27	(14.5)
Gambling problems <12 months						
No	1560	(97.9)	1272	(89.7)	146	(10.3)
Yes	33	(2.1)	23	(71.9)	9	(28.1)
Family member sent to/in jail <12 months						
No	1201	(74.9)	970	(90.0)	108	(10.0)
Yes	402	(25.1)	335	(87.2)	49	(12.8)
Difficulty getting a job <12 months						
No	987	(61.0)	821	(92.6)	66	(7.4)
Yes	631	(39.0)	498	(84.8)	89	(15.2)
In trouble with police <12 months						
No	1145	(82.4)	975	(89.9)	109	(10.1)
Yes	245	(17.6)	196	(82.7)	41	(17.3)

Overcrowding at home <12 months						
No	1477	(92.0)	1203	(89.9)	135	(10.1)
Yes	128	(8.0)	104	(84.6)	19	(15.4)
Witness to violence <12 months						
No	1341	(83.2)	1104	(90.9)	111	(9.1)
Yes	271	(16.8)	207	(81.2)	48	(18.8)
Hazardous alcohol use						
No	963	(55.2)	793	(87.7)	111	(12.3)
Yes	782	(44.8)	656	(89.1)	80	(10.9)
Smoking						
Non-smoker [c]	802	(45.5)	659	(93.3)	47	(6.7)
Current smoker	962	(54.5)	743	(84.6)	135	(15.4)
Amphetamine use						
No	1650	(92.5)	1334	(90.2)	145	(9.8)
Yes	133	(7.5)	94	(74.0)	33	(26.0)
Cannabis use						
No	1399	(76.2)	1128	(91.1)	110	(8.9)
Yes	437	(23.8)	340	(81.3)	78	(18.7)
Opiate use						
No	1689	(95.7)	1354	(89.4)	161	(10.6)
Yes	75	(4.3)	58	(80.6)	14	(19.4)
Cared for by someone else						
No	931	(84.4)	734	(87.6)	104	(12.4)
Yes	172	(15.6)	118	(84.9)	21	(15.1)
Children						
No	340	(31.5)	269	(82.8)	56	(17.2)
Yes	738	(68.5)	625	(89.5)	73	(10.5)
Single parent						
No	953	(77.7)	783	(86.8)	119	(13.2)
Yes	273	(22.3)	225	(87.2)	33	(12.8)
Community activity participation <12 months						
No	888	(57.0)	710	(86.7)	109	(13.3)
Yes	670	(43.0)	554	(92.2)	47	(7.8)
Exercise						
No	738	(41.0)	550	(85.7)	92	(14.3)
Yes	1060	(59.0)	877	(90.0)	97	(10.0)
Participation in sport						
No	1507	(83.9)	1183	(87.6)	168	(12.4)
Yes	290	(16.4)	249	(92.6)	20	(7.4)

Note: [a]Both represents Aboriginal and Torres Strait Islander; [b]Other includes unemployed, pension, studying, pair carer; [c]includes ex-smokers.

1. Islam, M.M.; Oni, H.T.; Lee, K.S.K.; Hayman, N.; Wilson, S.; Harrison, K., Hummerston, B.; Ivers, R.; Conigrave, K.M. Standardised alcohol screening in primary health care services targeting Aboriginal and Torres Strait Islander peoples in Australia. Addict Sci Clin Pract **2018**, *13*, 5.
2. Kowal, E.; Gunthorpe, W.; Bailie, R.S. Measuring emotional and social wellbeing in Aboriginal and Torres Strait Islander populations: an analysis of a Negative Life Events Scale. Int J Equity Health **2007**, *6*, 18.
